# Therapeutic efficacy of artemether–lumefantrine in North-Eastern states of India and prevalence of drug resistance-associated molecular markers

**DOI:** 10.1186/s12936-025-05338-1

**Published:** 2025-04-01

**Authors:** Shreelekha Dutta, Sri Krishna, Anup Kumar Vishwakarma, Sweta Mishra, Sushrikanta Khandai, Disphikha Goswami, Soni Kumari, Nazia Ali, Anil Kumar Verma, Kuldeep Singh, Aparup Das, Anup R. Anvikar, Praveen Kumar Bharti

**Affiliations:** 1 ICMR-National Institute of Research in Tribal Health, Jabalpur, Madhya Pradesh India; 2 ICMR-National Institute of Malaria Research, New Delhi, India

**Keywords:** Therapeutic efficacy, Artemether–lumefantrine, Antimalarial drug resistance, Molecular monitoring, North-East India

## Abstract

**Background:**

*Plasmodium falciparum* is the main cause of malaria in North-Eastern (NE) states of India. Artemether–lumefantrine (AL) was introduced as first-line therapy against uncomplicated *P. falciparum* cases in 2013 after the emergence of resistance to sulfadoxine–pyrimethamine. The aim of the study was to assess the therapeutic efficacy of AL and status of molecular markers in the circulating parasites.

**Methods:**

Therapeutic efficacy of AL was assessed in NE states as per World Health Organization guidelines. Patients with *P. falciparum* positive peripheral blood smear were enrolled and treated with AL and clinical and parasitological parameters were monitored over a 28-day follow-up period. Furthermore, the *pfmdr1*, *pfdhfr*, *pfdhps* and *pfk13* genes were amplified and sequenced for mutation analysis.

**Results:**

A total of 231 cases were enrolled and therapeutic efficacy was determined in 215 (93.1%) patients who completed their 28 days’ follow-up while 10 patients withdrew and 6 were lost to follow up during study. Overall 99.5% and 98.6% of adequate clinical and parasitological response was observed with and without PCR correction, respectively. Only three cases (1.4%) of late parasitological failure were observed in Mizoram site. One case of recrudescence and two cases of reinfection were detected by *msp1* and *msp2* genotyping. Mutation analysis showed the 15.8%, 100%, 90.5% mutants in *pfmdr1*, *pfdhfr* and *pfdhps* gene respectively and three non-synonymous mutations were also found in *pfk13*gene.

**Conclusions:**

This study reports that AL is efficacious against uncomplicated *P. falciparum* cases in NE states of India. However, prevalence of mutations in molecular marker associated with anti-malarial resistance (*pfmdr1*, *pfdhfr*, *pfdhps* and *pfk13*) gene indicate possible emergence of drug resistance. This is to underline the fact that the drug is efficacious for now, but rising mutations indicate that continuous monitoring is essential for effective treatment regime.

**Supplementary Information:**

The online version contains supplementary material available at 10.1186/s12936-025-05338-1.

## Background

Malaria is a vector borne parasitic disease caused by genus *Plasmodium*. In 2023, an estimated 263 million cases of malaria worldwide, with 94% cases from the African region and1.5% cases from the South-East Asian region [1]. India alone contributes to 50% of the malaria burden in the South East Asian region and India along with Indonesia accounted for 88% malaria deaths in this region. *Plasmodium falciparum* and *Plasmodium vivax* predominantly, cause malaria in India [2]. North-East (NE) Indian states along with Odisha, Chhattisgarh and Jharkhand contribute most of the *P. falciparum* cases in the country. Eight states, i.e. Assam, Arunachal Pradesh, Meghalaya, Mizoram, Nagaland, Sikkim, Manipur and Tripura, that constitute the NE India alone contribute to 18% of India’s *P. falciparum* cases [2].

North-eastern states of India, being a corridor to several other countries of South-east Asia (SEA), play an important role in transmission of drug resistant *P. falciparum* malaria into India. Emergence of chloroquine (CQ) resistance and sulfadoxine–pyrimethamine (SP) resistance were reported in NE for the first time in India, which has made it an epidemiologically important region in the transmission of resistant strains from SEA [3–5]. Consequently, artemether–lumefantrine (AL) was introduced as the new artemisinin-based combination therapy (ACT) against *P. falciparum* in NE Indian states in 2013 [6, 7]. Artemether is absorbed rapidly having a short half-life and gives a rapid reduction of parasite count while lumefantrine, the partner drug in this ACT is a lipophilic arylaminoalcohol that has low bioavailability [8, 9]. Further, lumefantrine is thought to inhibit haemozoin formation in the digestive vacuole (DV) of the erythrocytic-stage malaria parasite [10, 11].

Many mutations such as F446I, N548Y, N548I, M476I, M476V, Y493H, R539T, P553L, R561H and C580Y in *kelch 13* propeller gene are known to be associated with artemisinin resistance [12, 13]. Among these, total 13 mutations have been identified in India such as F446I, R561H, A481V, A675V, D702N in Arunachal Pradesh; A578S in Mizoram; G533A in Tripura; R539T, G625R, N672S, S549Y in West Bengal and M579T, N657H in Madhya Pradesh [14]. The mutation R539T along with G625R has been linked to delayed parasite clearance and high survival rates in eastern India [5].

Despite NE being a highly endemic region for malaria transmission, malaria intervention studies are challenging due to inaccessibility, difficult terrains and forested areas, administrative issues in the inter-state and international border areas, frequent flooding, poor roads and inadequate network connectivity further add to the challenge of field surveillance [15, 16]. Geography and demography of the region, predominance of *P. falciparum* over *P. vivax*, early emergence of drug resistance to anti-malarials, and the presence of highly anthropophillic vectors like *Anopheles baimaii* and *Anopheles minimus* in the region makes it different from the rest of India for malaria control and elimination. Periodic monitoring of therapeutic efficacy is recommended by World Health Organization (WHO) to control the malaria and avoid the spread of resistant parasite in the population. Therefore, therapeutic efficacy of AL for the treatment of uncomplicated *P. falciparum* malaria was assessed in NE states. Additionally, mutations in four different genes of *P. falciparum* (*mdr1, dhfr, dhps* and *k13*) that confer resistance to CQ, SP and artemisinin were analysed in field isolates to assess emerging resistance before it reaches deleterious levels. Mutation analysis of the *pfmdr1*gene was done to monitor the abundance of CQ sensitive while *pfdhfr* and *pfdhps* genes were selected to understand circulation of SP resistant strain in these states.

## Methods

### Study site

**Fig. 1:**
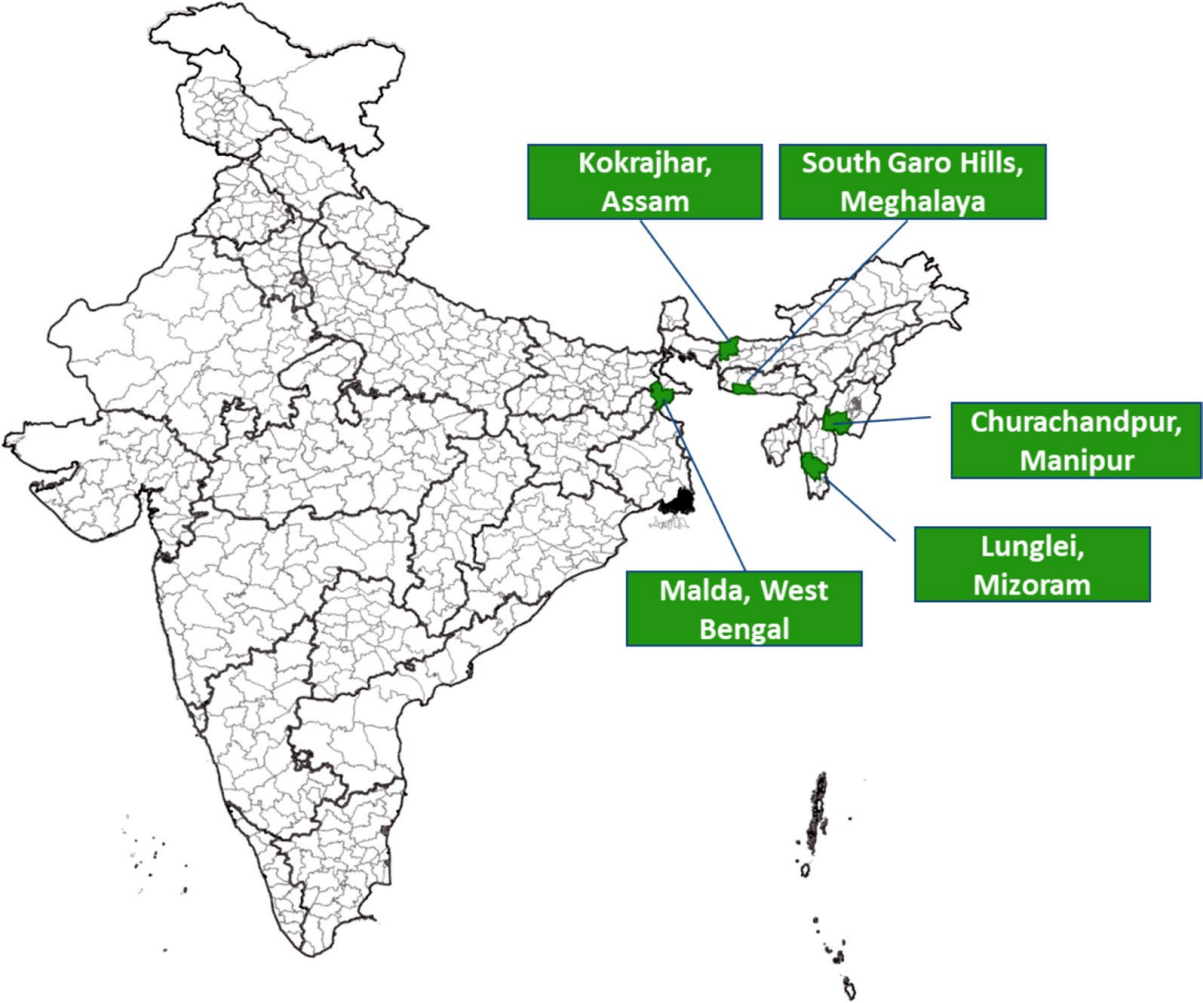
Map showing the study sites i.e. district Lunglei (Mizoram), district Kokrajhar (Assam), district South Garo Hills (Meghalaya), district Malda (West Bengal) and district Churachandpur (Manipur)

The selected study sites are Lunglei district (Mizoram), Kokrajhar district (Assam), South Garo-hills district (Meghalaya), Malda district (West Bengal) and Churchandpur district (Manipur) (Fig. 1)**.** The proposed districts have been decided in consultation with the national programme. These sites are located near international borders.

### Ethical statement

The study protocol for patient participation and collection of blood samples for laboratory testing was reviewed and approved by the institutional ethics committee of ICMR-National Institute of Research in Tribal Health (ICMR-NIRTH), Jabalpur. All study participants provided written informed consent prior to their participation according to Indian Council of Medical Research (ICMR), New Delhi, India guidelines. A copy of the consent form in the local language was also provided and explained to the patients or the parents/guardian of children.

### Screening of patients, enrolment and sample collection

Febrile patients aged between 1 and 60 years were screened for malaria parasite by microscopy and patients with confirmed uncomplicated mono *P. falciparum* infection were asked to participate in the study from period April- December 2019. Pregnancy test was performed on married female patients before enrollment into the study. Unmarried female, 12–18 years old (Menarche) were excluded from the study as request for pregnancy test and initiation of contraceptive was not acceptable in the local areas.

Patients with positive peripheral smear for *P. falciparum* malaria and fulfilling the enrollment criteria were enrolled in the study. The demographic information (age, gender, body temperature) were recorded. Two to three drops of finger prick blood were blotted on to 3 MM filter paper (Whatman International Ltd., Maidstone, UK) at the time of enrollment and during the follow-up for molecular study.

### Inclusion criteria

Symptomatic patients in the age group of 1–60 years and body weight > 5 kg diagnosed with mono-infection of uncomplicated *P. falciparum* malaria (detected by microscopy with parasitaemia in the range of 1000–100,000 asexual forms/μL and axillary temperature ≥ 37.5 °C) and willing to comply with the study protocol for the duration of the study were enrolled in the study [17].

### Exclusion criteria

Patients with signs of severe *P. falciparum* malaria or any other general danger signs and severe malnutrition; Mixed or mono infection other than *P. falciparum* species detected by microscopy; Febrile condition caused by disease other than malaria or other known underlying chronic or severe disease; Female patients with positive pregnancy test or breastfeeding; Patients who were unable to take medicine orally, had severe vomiting, decreased consciousness, inability to sit or stand, were all excluded [17].

### Anti-malarial treatment with ACT

Artemether–lumefantrine tablets were given orally according to body weight, twice a day over 3 days (0, 1, 2 day) as per national guideline [18] under supervision of a clinician. The day patient was enrolled for study was recorded as zero day. Clinical and parasitological parameters were monitored over a 28-day follow-up period (0, 1, 2, 3, 7, 14, 28 day) and therapeutic efficacy was determined following WHO standard protocol [17].

### Microscopic blood examinations

Parasite counts were done on JSB-stained thick blood films and the numbers of parasites per 200 white blood cells (WBCs) were counted by light microscopy. Parasite density, expressed as the number of asexual parasites per μL of blood, was calculated by dividing the number of asexual parasites by the number of WBCs counted, and then multiplying it by an assumed WBC density (typically 6000 per μL). When the number of asexual parasites was fewer than 100 per 200 WBCs in follow-up smears, counting was done against at least 500 WBCs. A blood slide sample was considered negative when examination of 1000 WBCs or 100 fields containing at least ten WBCs per field revealed no asexual parasites. The presence of gametocytes on the day the patient was enrolled or on the day of follow-up was also recorded.

### Quality assurance of microscopy

Blood smears of enrolled patients, including follow-up smears, were examined by two independent microscopists (one at each study site and another at the ICMR-NIRTH Laboratory, Jabalpur). If any discordance was found, a third reading was performed by another senior microscopist who was unaware of the result. Quantification of parasitaemia was also performed by two independent microscopist with a third reading performed by a senior microscopist, if the difference between the first two readings varied by more than 25%. The result of third microscopist matching with any one of the first or second microscopist was considered as final result. Each reader was blinded to the result of other reader.

### Genomic DNA extraction and parasite genotyping

Genomic DNA was isolated by QIAamp DNA blood mini kit as per the manufacturer’s instructions (Qiagen, CA, USA) and stored at − 20 °C till further use. Samples were analyzed for *Plasmodium* species by species specific nested PCR as per previous protocol [19]. Amplification of *msp1* and *msp2* genes was performed in as per previously published protocol [20]. Recrudescence was considered as at least one identical allele for both markers (*msp1* and *msp2*) in the samples of Day 0 and day of parasite observed. Reinfection was defined when all alleles for at least one of the markers differed between the two samples.

The*pfmdr1* gene region covering codons 47–332, *pfdhfr* gene spanning codons 15–170 and *pfdhps* gene spanning codons 425–640 were amplified using nested PCR and the details of nested PCR primers and cycling conditions are given in the supplementary table. In brief, primary PCR was performed in a volume of 25 μL with 0.2 U of Taq polymerase enzyme (Invitrogen, life technologies), 0.2 mM each dNTPs, 1 μM each primer and 1.5 mM MgCl_2_. Furthermore, the propeller region of *pfk13* gene from codon 427 to 709 was amplified and sequenced for tracking the emergence and spread of artemisinin resistance in *P. falciparum*.

### Nucleotide sequencing

The PCR product was used with the ABI Big dye Terminator cycle sequencing kit Version 3.1 (Applied Biosystems, USA) for sequencing PCR. The sequencing PCR was performed in a volume of 10 μL with 0.5 μL of ready reaction mix, 1.6 pmol of gene specific primer and 5X sequencing buffer as per manufacturer’s protocol. These cycle sequencing PCR products were purified and sequenced with 3730 DNA analyzer (Applied Biosystems, USA). The obtained sequences were aligned and analysed using Bio Edit Sequence Alignment editor v.7.0.5.2 software and GeneDoc software version 2.7.000 [21].

### Statistical analysis

All the data was entered into Microsoft excel sheet and analysed. Further analysis of statistical parameters such as haplotype and nucleotide diversity (θ & π), Tajima’s D test were estimated using DnaSPv6 software version 6.12.03 [22]. In addition, to determine the non-random association between SNPs in *pfmdr1*, *pfdhfr* and *pfdhps* genes among parasite population linkage dis-equilibrium analysis was performed using Haploview software [23]. Graphs were plotted using GraphPad prism software.

## Results

### Study participants and clinical response

**Fig. 2:**
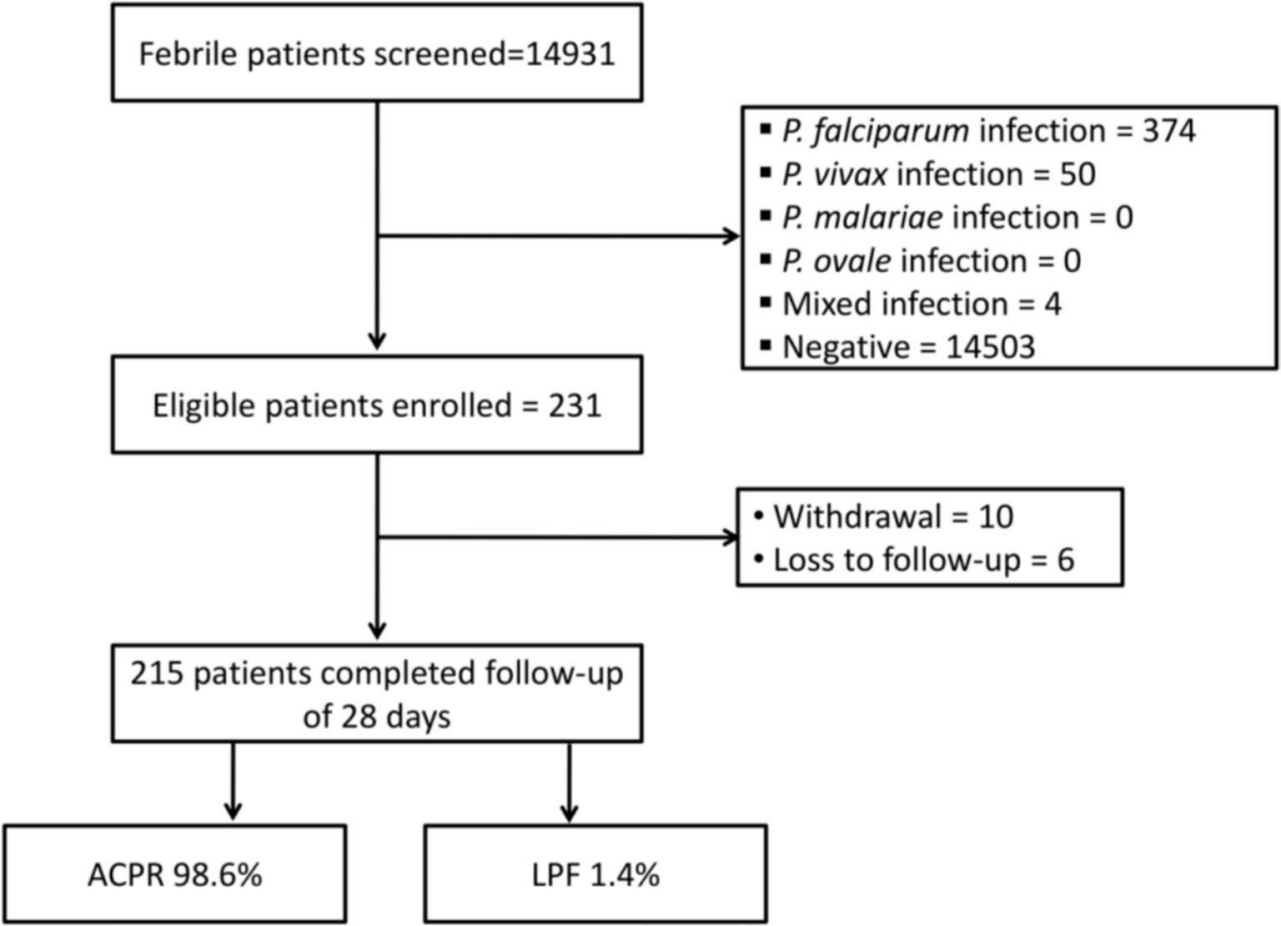
Schematic representation of overall patient screening, enrolment and follow-up

Overall, a total of 14,931 patients in the age group of 1 to 60 years were screened for malaria parasite during the study period. Malaria positivity rate was 2.9% (428/14931) with 87.4% (374/428) *P. falciparum* mono infection. Further, A total 11.68% (50/428) case of *P. vivax* and 0.9% (4/428) mix infection of *P. falciparum* and *P. vivax* was also recorded (Fig. 2).

Out of 374 mono *P. falciparum* cases, a total 231 cases who fulfilled the enrolment criteria as well as consented for the study were enrolled. Therapeutic efficacy was determined in 215 (93.1%) patients who had completed the 28 days follow-up while 16 patients either withdrew from the study or were lost to follow up due to various reasons (Fig. 2). Out of 16, a total of 10 patients were withdrawn from the study as they were unable to complete the treatment due to persistent vomiting or failure to attend the scheduled visits during the first 3 days (Day 0 to Day 2). Rest 6 patients were lost to follow-up as they could not attend scheduled visits and unable to trace because of remote area and migration of population for their earning and livelihood and thus excluded from the final analysis.

Background characteristics (age, sex, parasitaemia) of the enrolled patients are summarized in Table 1. Most of the patients (47.2%) were in the age group of 5–15 years old followed by adults (36.8%) and the rest (16%) were less than 5 years of age. Similar trend in patient’s age was observed at each site. Overall, 55% patients were male and 45% were female and similarly number of male patients was more than female patients at each site except in Meghalaya. Geometric Mean (95% CI) parasite density at baseline was recorded highest at study site in Mizoram (5474) followed by Meghalaya (3567), Assam (3266) and lowest at study site in West Bengal (2242).

**Table 1:** Summary statistics of the enrolled subjects in four study sites

	MZ (n = 88)	Assam (n = 75)	MG (n = 66)	WB (n = 2)	Total (n = 231)
Age (years)					
Mean	16.3	16.6	16.1	26.5	18.875
SD	14.6	14.8	13	12	13.6
Min	1	1	2	18	1
Max	60	50	60	35	60
Age group (years)					
Under 5 (%)	16 (18.2)	13 (17.3)	8 (12.1)	0	37 (16.0)
5–15(%)	40 (45.5)	38 (50.7)	31 (46.9)	0	109 (47.2)
Adult (%)	32 (36.4)	24 (32.0)	27 (40.9)	2 (100)	85 (36.8)
Sex					
Male (%)	55 (62.5)	41 (54.67)	29 (43.9)	2 (100)	127 (55.0)
Female (%)	33 (37.5)	34 (45.3)	37 (56.1)	0 (0.0)	104 (45.0)
Parasite density/µL					
Geometric Mean	5474	3266	3567	2242	3637.25
Max	96,000	68,955	91,057	3470	96,000
Min	1020	1270	1510	1448	1020

*MZ* Mizoram, *MG* Meghalaya, *WB* West Bengal

The total enrolment, site wise number of enrollment and progress of follow-up at each study sites are summarized in Table 2. Adequate clinical and parasitological response (ACPR) was observed in 98.6% of cases with only three cases (1.4%) of late parasitological failure (LPF).

**Table 2:** Site wise summary of patient enrollment, follow up status, treatment outcomes and parasite clearance time

Variables	Study sites					
	MZ	Assam	MG	WB	MN	Total
No. screened	1120	2948	7890	2898	75	14,931
No. enrolled	88	75	66	2	0	231
Withdrawal	6	2	2	0	0	10
Loss to follow-up	4	1	1	0	0	6
Therapeutic efficacy without PCR correction						
Early treatment failure	0	0	0	0	0	0
Late clinical failure	0	0	0	0	0	0
Late parasitological failure	3	0	0	0	0	3
Adequate clinical & parasitological response (%)	75 (96.2)	72 (100)	6 3(100)	2 (100)	–	212 (98.6)
Therapeutic efficacy with PCR correction						
Early treatment failure	0	0	0	0	0	0
Late clinical failure	0	0	0	0	0	0
Late parasitological failure	1	0	0	0	0	1
Adequate clinical & parasitological response (%)	75 (98.7)	72 (100)	63 (100)	2 (100)	–	212 (99.5)
*Pf* recrudescence	1	0	0	0	0	1
*Pf* re-infection	2	0	0	0	0	2
Mixed with Pf recrudescence	0	0	0	0	0	0
PCR negative	0	0	0	0	0	0
Parasite clearance time (n = 226)						
≤ 24 h	65 (83.3)	53 (73.6)	50 (79.4)	1 (50.0)	0 (0.0)	169 (74.8)
> 24-48 h	17 (21.8)	20 (27.8)	14 (22.2)	1 (50.0)	0 (0.0)	52 (23.0)
> 48-72 h	1 (1.3)	0 (0.0)	1 (1.6)	0 (0.0)	0 (0.0)	2 (0.9)
> 72 h	3 (3.8)	0 (0.0)	0 (0.0)	0 (0.0)	0 (0.0)	3 (1.3)

*WB* West Bengal, *MZ* Mizoram, *MG* Meghalaya, *MN* Manipur

No cases of early treatment failure (ETF) or late clinical failure (LCF) was observed in this study. In most of the patients (78.6%) parasitaemia was cleared within ≥ 24 h. The parasite clearance time was more in the Mizoram and Meghalaya in comparison to Assam and West Bengal (Table 2).

#### Molecular analysis of samples with LPF

All three samples from 0 day and day of LPF were analysed and found positive for *P. falciparum* by species specific nested PCR. To understand whether treatment failures were recrudescence or reinfection, *P. falciparum msp1* and *msp2* genes were amplified and sequenced from samples of 0 day and day of LPF. Out of three cases, one contains the same allele for both the genes (*msp1* and *msp2*) among 0 day and day of LPF (recrudescence), whereas other two patients had re-infection in which *msp1* and *msp2* allele were different at the time of LPF.

### Molecular analysis of genes associated with anti-malarial drug resistance

#### Analysis of pfmdr1 gene

**Fig. 3:**
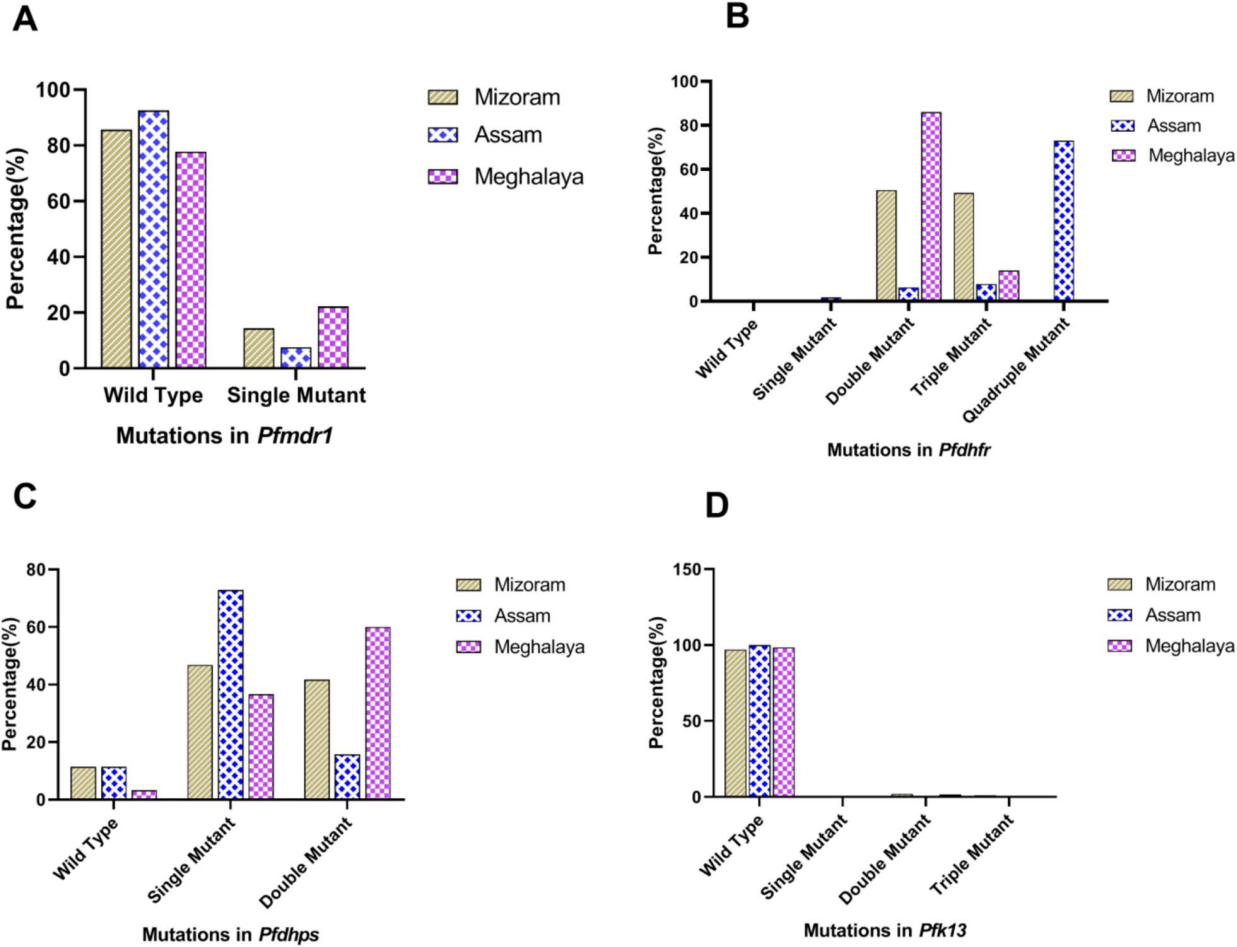
Graphical representation of mutations in *Pfmdr1* (**A**), *Pfdhfr* (**B**), *Pfdhps* (**C**) and *Pfk13* (**D**) in Mizoram, Assam and Meghalaya (West Bengal was not shown in figure due to low sample size)

A total of 215 isolates from the four study sites were successfully amplified and analysed for *pfmdr1* gene. Out of 215 samples, 15.8% were mutant. Single mutant at Y184F was found in 13 samples from Mizoram and 13 samples from Meghalaya and one sample from West Bengal. Single mutant at N86Y was found in four samples from Assam, one sample from Mizoram and a double mutation at N86Y, Y184F was observed in one sample from West Bengal (Fig. 3A, Table 3).

**Table 3:** Site-wise distribution of non-synonymous mutant haplotypes in *pfmdr1*, *pfdhfr*, *pfdhps*and *pfk13* genes

Gene	Haplotypes	MZ n (%)	Assam n (%)	MG n (%)	WB n (%)	Total
*pfmdr1*	Wild type	83 (85.6)	49 (92.5)	49 (77.8)	0.0	181
	N86Y	1 (1.0)	4 (7.5)	0.0	0.0	5
	Y184F	13 (13.4)	0.0	13 (20.6)	1 (50.0)	27
	V216I	0.0	0.0	1 (1.6)	0.0	1
	N86Y, Y184F	0.0	0.0	0.0	1 (50.0)	1
	Total (N)	97	53	63	2	215
*pfdhfr*	Wild type	0.0	0.0	0.0	0.0	0.0
	S108N	0.0	8 (12.7)	0.0	0.0	8
	C59R, S108N	45 (50.6)	4 (6.3)	49 (86.0)	0.0	98
	N51I, C59R, S108N	44 (49.4)	1 (1.6)	8 (14.0)	0.0	53
	C59R, S108N, I164L	0.0	2 (3.2)	0.0	0.0	2
	N51I, S108N, I164L	0.0	2 (3.2)	0.0	0.0	2
	N51I, C59R, S108N, I164L	0.0	46 (73.0)	0.0	0.0	46
	Total (N)	89	63	57	0.0	209
*pfdhps*	Wild type	9 (11.4)	8 (11.4)	2 (3.3)	1 (50.0)	20
	G437A	5 (6.3)	10 (14.3)	11 (18.3)	1 (50.0)	27
	K540E	19 (24.1)	39 (55.7)	5 (8.3)	0.0	63
	A581G	13 (16.5)	2 (2.9)	6 (10.0)	0.0	21
	S436A_,_ K540E	31 (39.2)	11 (15.7)	35 (58.3)	0.0	77
	G437A, K540E	0.0	0.0	1 (1.7)	0.0	1
	K540E_,_ A581G	2 (2.5)	0.0	0.0	0.0	2
	Total (N)	79	70	60	2	211
*pfk13*	Wild type	100 (97.1)	66 (100)	61( 98.4)	1 (100)	228
	M579T, N657H	2 (1.9)	0	1 (1.6)	0	3
	I543M, M579T, N657H	1 (1.0)	0	0	0	1
	Total (N)	103	66	62	1	232

**Fig. 4:**
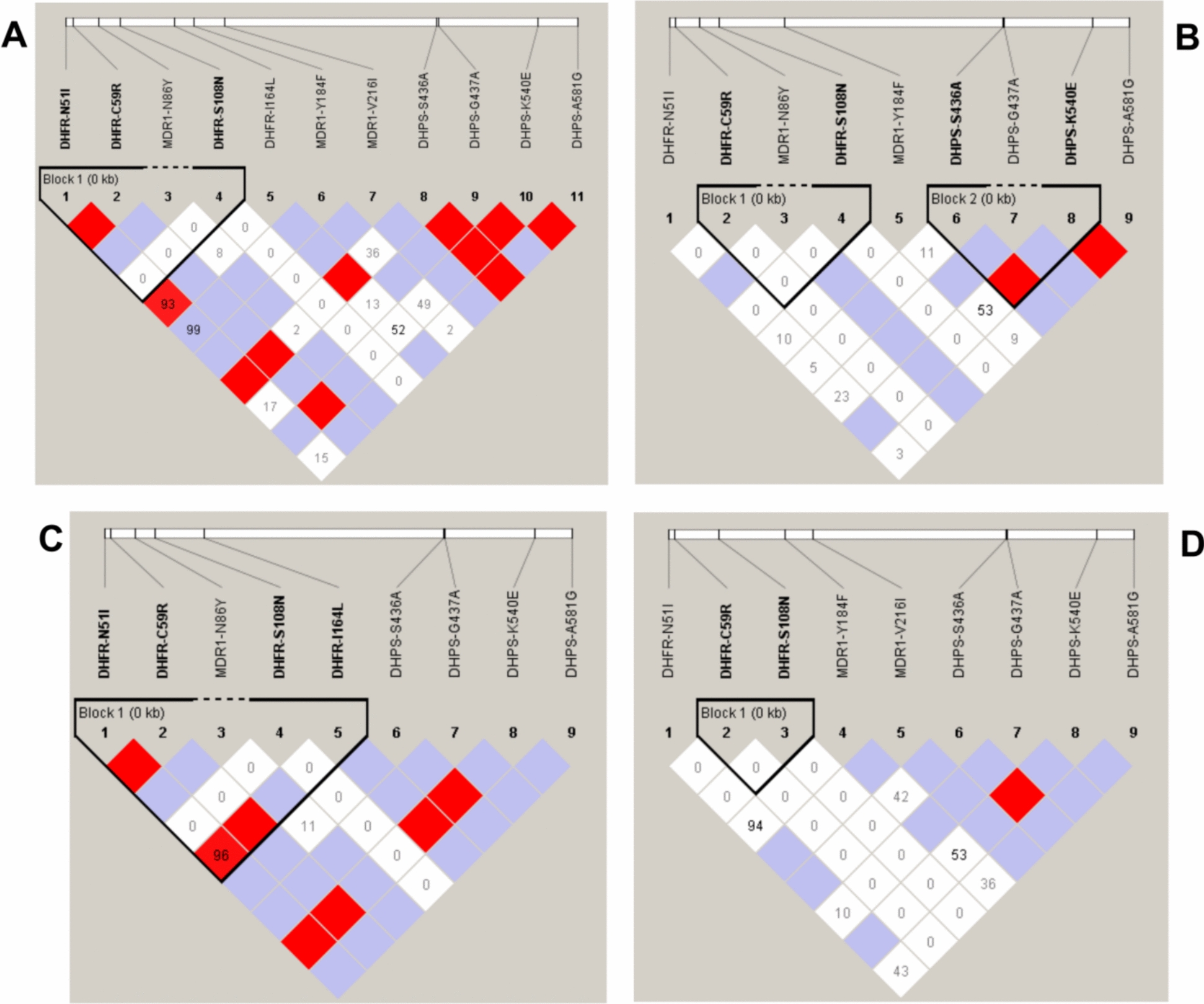
Linkage Disequilibrium of Single Nucleotide Polymorphism (SNPs) in *Pfmdr1*, *Pfdhfr*, *Pfdhps* genes among parasite population showing LD plot of SNPs in all three states (Mizoram, Assam and Meghalaya) (**A**) and Mizoram (**B**); Assam (**C**); Meghalaya (**D**)

Sequence representing *pfmdr1* haplotypes from each state were submitted to NCBI GenBank database. Figure 4 demonstrates the graphical representation of site-wise distribution of genetic mutations observed in the samples from various states. Analysis of LD plot was not showing any strong linkage Disequilibrium between SNPs in *pfmdr1* gene among parasite population of all three states (Fig. 4).

#### Analysis of *pfdhfr* and *pfdhps* gene

A total of 209 isolates were successfully amplified and analysed for *pfdhfr* gene respectively which harbored either single, double or a triple mutation. Polymorphism was observed in four (N51I, C59R, S108N and I164L) out of the five *pfdhfr* mutations previously known to confer resistance to the partner drug, pyrimethamine in ACT. Among the samples collected from Mizoram, 50.6% showed double mutants whereas 49.4% showed triple mutants for *pfdhfr* gene. In samples from Assam, 73% were quadruple mutant, 7.9% were triple mutant, 6.3% double mutant and 12.7% were single mutant for *pfdhfr* gene. In samples from Meghalaya, 86% were double mutants and 14% were triple mutants (Fig. 3B, Table 3) for *pfdhfr* gene. Overall, double-mutant haplotype C59R, S108N was the most prevalent (n = 98), followed by_,_ a triple-mutant haplotype (n = 57) respectively. Quadruple mutant haplotype was observed in Assam only (Table 3). Sequence representing *pfdhfr* haplotypes from each state were submitted to NCBI GenBank database. One block containing SNPs N51I, C59R and S108N was identified in *pfdhfr* gene and darkness of the block colour showed linkage disequilibrium between them (Fig. 4A). State wise analysis also showed the formation of one block in each state but SNPs were different such as C59R, S108N in Mizoram (Fig. 4B); N51I, C59R, S108N and I164L) in Assam (Fig. 4C); C59R and S108N in Meghalaya (Fig. 4D).

Subsequently, the samples were analysed for *pfdhps* gene which covers 430–601 codon. A total of 211 samples were sequenced which showed six, five and six haplotypes in samples from Mizoram, Assam and Meghalaya, respectively with the highest haplotype diversity of 0.753 in samples collected from Mizoram (Fig. 3C, Table 4). Of the 211 samples analysed, 90.5% (n = 191) were mutant type and the rest 9.5% (n = 20) were wild type. Of the five *pfdhps* mutations (S436F/A, G437A, K540E, A581G and A613S/T) known to be involved in ACT (AS + SP)’s partner drug, sulfadoxine resistance, the study population depicted mutations in four codons: S436F/A, G437A, K540E and A581G. Double mutant (S436A, K540E) was the most prevalent haplotype, followed by single mutant K540E, A581G, G437Aand double mutant (G437A, K540E) in the study population (Fig. 3C). The site-wise distribution of the mutants and the polymorphism analysis is depicted in Tables 3 and 4. Linkage Disequilibrium analysis showed one block containing two SNPs in *pfdhps* gene S436A and K540E in Mizoram only (Fig. 4B).

**Table 4:** Site-wise Diversity analysis of *pfmdr1*, *pfdhfr*, *pfdhps* and *pfk13* genes

Genes	Study Site	Number of mutations		Number of haplotypes	Haplotype diversity	Nucleotide diversity		Tajima's D
		Non-synonymous	Synonymous			π	θ	
*pfmdr1*	MZ	2	0	3	0.252	0.00039	0.389	− 0.53100
	AS	1	1	3	0.403	0.00065	0.441	− 0.04435
	MG	2	2	5	0.447	0.00087	0.849	− 0.69073
*pfdhfr*	MZ	3	0	2	0.506	0.00074	0.198	1.83032
	AS	4	0	6	0.452	0.00141	0.637	0.98350
	MG	3	0	2	0.246	0.00036	0.217	0.17238
*pfdhps*	MZ	4	1	8	0.753	0.00236	1.012	0.90596
	AS	4	0	5	0.628	0.00160	0.830	0.34978
	MG	4	1	7	0.621	0.00265	1.072	1.14582
*pfk13*	MZ	3	0	3	0.057	0.00017	0.576	− 1.37085
	AS	0	0	1	0.000	0.0000	0.000	NA
	MG	2	1	2	0.032	0.00013	0.639	− 1.67192

#### Analysis of pfk13 gene

Analysis of *pfk13* gene showed two double mutant M579T, N657H and one triple mutant I543M, M579T, N657H in Mizoram. In Assam only one double mutant M579T, N657H in Meghalaya while no mutation was observed in Assam (Fig. 3D, Table 3). Analysis of DNA polymorphism of *pfmdr1, pfdhfr*, *pfdhps* and *pfk13* genes are shown in Table 4.

## Discussion

Anti-malarial drug resistance in *P. falciparum* is a cause of major concern in India’s efforts to eliminate malaria by 2030. The efforts to combat drug resistance in North-East India are of paramount significance since cases of CQ and SP resistance were first reported in NE India and the origin of this resistance can be traced back to SEA [3, 4]. Similarly, delayed parasite clearance in *P. falciparum* malaria patient and reduced susceptibility to artesunate was first reported from Cambodia that belongs to SEA region in 2008 and 2009 respectively [24, 25]. It is also assumed that artemisinin resistant strain of *P. falciparum* might have spread from Cambodia to NE region of India through Thailand and Myanmar [26, 27]. With increasing trends of partner drug resistance markers, there is a need for continuous monitoring and tracking of emergence of any mutations in these markers.

In the present study the ACPR was 100% at all the three sites except Mizoram where three cases of LPF were found, although these cases were related to recrudescence (n = 1) and reinfection (n = 2) after PCR correction. In earlier studies, high efficacy of AL was observed with cases of LPF from only Bastar, Chhattisgarh [28, 29]. Similarly, another study has shown the high cure rate of AL in Sub-Saharan Africa [30]. In a recent study from North-Eastern states of India and Tanzania, AL was found highly effective in treatment of *P. falciparum* malaria [31, 32]. In this study, polymorphism in *pfmdr1, pfdhfr, pfdhps* was assessed to track emergence of resistance to the partner drug in ACT, and *pfk13* gene to track emergence of artemisinin resistance. Analysis of *pfmdr1* SNPs showed presence of mutation at N86Y, Y184F and a double mutation at N86Y + Y184F. Amino acid mutations N86Y, Y184F, and D1246Y have been associated with reduced susceptibility to lumefantrine [33]. In contrast, an in-vitro study on engineered parasite with mutation N86Y and Y184F has indicated the role of N86Y in increasing sensitivity to AL, mefloquine and dihydroartemisin in while Y184F has limited impact [34]. Similarly, in another study, wild type Y184 was positively selected by AL and N86, D1246 was associated with decrease sensitivity to lumefantrine [35, 36]. In the present study, 7.5%and 1.0% of the samples had haplotypes (N86Y) in Assam and Mizoram, respectively, while Y184F was observed in Mizoram (13.4%) and Meghalaya (20.6%) only. Thus indicating a regular need to monitor these markers of *pfmdr1* gene associated with tolerance to arylaminoalcohol drug/lumefantrine. Tolerant parasites, which are in the intermediate stage between sensitive and resistant, are eliminated by the high bioavailability of drug during treatment but can survive residual lumefantrine concentrations and proliferate in the blood earlier than the sensitive parasite. Consequently, increasing the selection pressure on resistant parasites.

Further, high prevalence of 100% *pfdhfr* mutation (N51I, C59R, S108N and I164L) and 91.5% *pfdhps* mutations (S436A, G437A, K540E, A581G) were observed in the study population. A significant predictor of clinical treatment failure is a combined quintuple mutant of *pfdhps* gene (G437A and K540E) and *pfdhfr* gene (N51I, C59R and S108N) are already known [30]. Single point mutations in these genes could be indicative of early sign of the improper action of the drugs, while double mutation may indicate a decreased sensitivity of parasite to the drug and multiple mutations (three or more) raise concerns towards early indication of a progression to drug resistance [37, 38]. It is noteworthy to mention that 27.3% of the study population had triple mutation at codon 51, 59, 108 or 164, while quadruple mutation was observed in Assam only in the *pfdhfr* gene. Moreover, mutations observed in *pfdhps* gene were either single or double mutants. The quadruple mutation in *pfdhfr* and double and triple mutations in *pfdhps* also have been reported by other studies in India [39–42].

With artemisinin resistance spreading geographically from Cambodia to Greater Mekong sub-region, India needs a strict surveillance on the markers associated with it. So far, India has not reported any ineffectiveness of artemisinin derivatives, however presence of non-synonymous mutations in the propeller region of *pfk13* are a sign of worry for malaria eliminations efforts. Kelch 13 structurally dimeric (Protein Data Bank ID: 4YY8), is a member of the Kelch-like superfamily [43]. Studies have been carried out previously to analyse for the distribution of artemisinin resistance mutations and its dimerization, but these analyses did not establish any structural association with the proposed artemisinin failure phenotype [44, 45]. Present study showed 3 non-synonymous mutations at I543M, M579T and N657H in the propeller region of *pfk13* gene. M579T and N657H, double mutation was observed in two samples from Mizoram and one sample from Meghalaya. Triple mutant (I543M, M579T, N657H) was observed in one sample in Mizoram only. Of these mutations, double mutant (M579T and N657H) have been previously reported from Madhya Pradesh, India [28, 46] while Q613H has been reported from Chhattisgarh, India [29]. Among the known mutation in *pfk13* causing artemisinin resistance, R539T and one novel mutation G625R associated with artemisinin resistance also has been recorded from West Bengal, India [13]. Additionally, mutation at 543 codon is a validated artemisinin resistance mutation [47]. This study reports high prevalence of molecular marker associated with anti-malarial resistance in *pfmdr1*, *pfdhfr*, *pfdhps* and *pfk13* gene, however, no effect on clinical outcome of the drug was observed. This is to underline the fact that the drug is efficacious for now, but high prevalence of non-synonymous mutations is an early indication of emerging resistance.

## Conclusion

The clinical outcome of 99% cure rate is indicative of the efficacy of the drug (AL) in the North-East India as the first-line of therapy for treatment of uncomplicated *P. falciparum* malaria according to the National Drug Policy. However, understanding the molecular basis of progression from susceptibility to resistance via tolerance against this combination is fundamental to establishment of measures to safeguard the development of resistance in *P. falciparum*. Regular surveillance and monitoring of molecular markers is required for periodic assessment of therapeutic intervention until the elimination of disease is achieved.

## Supplementary Information

Supplementary Material 1.

## Data Availability

No datasets were generated or analysed during the current study.
