# Supplementary material for: Therapeutic efficacy of artemether–lumefantrine in North-Eastern states of India and prevalence of drug resistance-associated molecular markers
Source: Malar J. 2025 Apr 1;24:106. doi: 10.1186/s12936-025-05338-1 (PMC11959953; doi:10.1186/s12936-025-05338-1)
Supplement: Supplementary file 1 — Supplementary Material 1. [file 12936_2025_5338_MOESM1_ESM.docx]

**Supplemental Data**

**Supplementary table1:** Primers and profiles used for PCR amplification of the *pfdhfr, pfdhps* and *pfk13* genes

| **Gene** | **Primer name** | **Primer sequences (5’-3’)** | **Annealing**  **Temp**  **(ºC)** | **No. of Cycle** | **Product size**  **(bp)** |
| --- | --- | --- | --- | --- | --- |
| ***pfdhfr*** | P1-F | TTTATATTTTCTCCTTTTTA | 45 | 40 | 742 |
|  | P2-R | CATTTTATTATTATTCGTTTTCT |  |  |  |
|  | P3-R2 | ACAGAAATAATTTGATACTCA | 45 | 35 | 542 |
| ***pfdhps*** | P1-F | CCATTCCTCATGTGTATACAACAC | 55 | 35 | 1167 |
|  | P2-R | CTTGGTCTATTTTTGTTAAAACATCC |  |  |  |
|  | P3-nF | TGGAATATTAAATGTTAATTATGA | 50 | 30 | 735 |
|  | P4-nR | TTTTCATTTTGTTGTTCATCATGT |  |  |  |
| ***pfk13*** | K13-1 | CGGAGTGACCAAATCTGGGA | 60 | 40 | 2097 |
|  | K13-4 | GGGAATCTGGTGGTAACAGC |  |  |  |
|  | K13-2 | GCCAAGCTGCCATTCATTTG | 60 | 35 | 849 |
|  | K13-3 | GCCTTGTTGAAAGAAGCAGA |  |  |  |

***pfdhfr*:** *Plasmodium falciparumdihydrofolate reductase,* ***pfdhps:*** *Plasmodium falciparum dihydropteroate synthase*, ***pfk13*:** *Plasmodium falciparum kelch13*, **Temp:** temperature, **bp:** base pair.
